# Cardiopulmonary Exercise Test in the Evaluation of Heart Transplant Candidates with Atrial Fibrillation

**DOI:** 10.36660/abc.20200051

**Published:** 2020-02

**Authors:** Miguel Mendes

**Affiliations:** CHLO - Hospital de Santa Cruz, Carnaxide - Portugal

**Keywords:** Heart Failure, Atrial Fibrillation, Heart Transplantation, Patient Selection, Oxygen Consumption, Exercise Test

Antonio Valentim Gonçalves et al.,^1^ authors of the original article “Prognostic Prediction of the Cardiopulmonary Exercise Test Parameters in Patients with Heart Failure and Atrial Fibrillation”,^1^ published in this issue of *Arquivos Brasileiros de Cardiologia*, intended to evaluate whether the cutoff points of two parameters of the cardiopulmonary exercise test (CPET), routinely used in the selection of patients for heart transplant (HT), would also be efficient in the presence of permanent or persistent atrial fibrillation (AF) in patients with heart failure with reduced ejection fraction (HFREF).

In their work, the authors assessed whether the study primary endpoint was reached in the presence of two recommendations of the International Society for Heart and Lung Transplantation (ISHLT) guideline:^2^ 1) peak oxygen consumption (pVO_2_) ≤ 12 (under betablocker therapy) - BB) or 14 mL/Kg/min (in the absence of BB) and, 2) slope of ventilation (VE) / carbon dioxide elimination (VCO_2_) > 35, when the respiratory exchange ratio (RER) during the exercise is < 1.05.

This study included 274 consecutive patients with left ventricular ejection fraction (LVEF) < 40%, from a single center, assessed by CPET, of which 51 were in AF and 223 in sinus rhythm (SR). The primary endpoint [HT or cardiac death (CD)] was observed in 17.6% of patients with AF and 8.1% of patients in SR (p < 0.0038).

In the context of AF, the VO_2_-related cutoff point (with or without BB) performed very well, with a positive predictive value (PPV) of 100% and a negative predictive value (NPV) of 95.5%. In contrast, the VE/VCO_2_ slope cutoff point was found to have a PPV of 33.8% and a NPV of 92.3%.

In the group of patients in SR, the results of the cutoff point related to pVO_2_ were lower, with a PPV of 38.5% and a NPV of 94.3%, similar to the cutoff point of the VE/VCO_2_ slope, with a PPV of 29.8% and a NPV of 98.3%.

They concluded that the current cutoff points accurately stratify patients in AF, corroborating the initial hypothesis of their research.

To the best of my knowledge, this is the first study that specifically assessed the application of the ISHLT criteria for the selection of patients with AF and HFREF for HT. The study is valuable for having assessed the application of these criteria in this group that has a significant dimension in heart failure (HF) clinics.

## Clinical application of the study findings

The main conclusions of the article are as follows:

1. The two ISHLT criteria were better suited to patients with AF than to those in SR.
2. In the context of AF, the performance of the peak VO_2_ criterion ≤ 12 or 14 mL/Kg/min, depending on whether or not the patient was under betablocker medication, has a much higher value than the VE/VCO_2_ slope.
3. In patients in SR, either of the two criteria (peak VO_2_ and VE/VCO_2_ slope > 35) have a low PPV (< 40%) and high NPV (> 90%); thus, they are more suitable to identify patients who do not need HT.

It seems logical that patients in AF, with LVEF < 40%, have a lower functional capacity than those in SR, because the AF reduces the maximum cardiac output by a percentage of not less than 25%. On the other hand, many of these patients have advanced HF,^3,4^ with less capacity to extract oxygen at the muscle level, as a result of the muscular atrophy caused by inactivity and the myopathy inherent to HF. As pVO_2_ is related not only to the cardiac output at the level of maximum effort, but also to the oxygen extraction capacity at the peripheral level, it is easy to understand why they have decreased pVO_2_.

It would have been interesting also to evaluate the criterion of pVO_2_ < 50% of the predicted maximum, in individuals under the age of 50 years or of the female gender, which was classified as class IIa, [level of evidence (LE) B], higher than the criterion VE/VCO_2_ slope, which was rated as class IIb (LE C). The criterion VE/VCO_2_ slope is indicated by the ISHLT for alternative use when a respiratory rate > 1.05 is not obtained during the exercise period.

The inefficient performance of the criteria used in the SR group, which obtained a PPV < 40%, is surprising. Part of the explanation may be related to the presence of 40% of women in the SR group, compared to 27.5% in the AF group (although with p < 0.087). Indeed, it has been shown that women have a better prognosis, despite having significantly lower pVO_2_ values than men.^5^

## The ISHLT criteria for risk stratification in HFREF

The 2016 ISHLT guideline^2^ for placing patients on a HT list was conservative and generally maintained the recommendations of 2006. It included once more a recommendation (class I, LE B) confirming the suitability of the pVO_2_ generic cutoff for patients with a cardiac resynchronization device following the COMPANION study and recommended using the prognostic scores [Heart Failure Survival Score (HFSS) and Seattle Heart Failure Model (SHFM)] together with the CPET parameters (class IIb, LE B).

Regarding the pVO_2_, it maintained the cutoff points pVO_2_≤ 12 (under BB therapy) and ≤ 14 mL/Kg/min (intolerant to BB therapy) as class I (LE B) recommendations. It considers reasonable to use as a cutoff point a pVO_2_ value < 50% of the maximum predicted in patients under the age of 50 years and in females, assigning it a IIa classification, LE B.

It recommends using the criterion VE/VCO_2_ slope > 35 only in cases of submaximal CPET, i.e., when the respiratory exchange ratio (RER) is < 1.05 at peak effort (class IIb, LE C).

Guazzi et al.^6^ in 2012 considered that mortality would be > 50%, between 1 and 4 years, if the criteria VE/VCO_2_ slope ≥ 45, pVO_2_ < 10.0 mL/Kg/min, and ventilatory oscillations (VO),^7^ the expired CO_2_ pressure (P_ET_CO_2_) < 33 mmHg at rest and with an increase of less than 3 mmHg during exercise were present. In addition to the recommendation of using stricter criteria in pVO_2_ and especially in the VE/VCO_2_ slope, Guazzi et al.^6^ introduced two new parameters in the assessment: the oscillatory breathing (OB) and P_ET_CO_2_. Before this publication, other authors, including Ferreira et al.,^8^ defined higher cutoff points for the VE/VCO_2_ slope. In this article, a cutoff point of 43 was defined, which is much stricter and more discriminative than the ISHLT criterion.

In 2016, Malhotra et al.^9^ demonstrated that patients with HFREF with pVO_2_ < 12 or 14 mL/Kg/min (with or without BB), VE/VCO_2_ slope > 36, OB, oxygen uptake efficiency below 1.4, reaching systolic pressure value < 120 mmHg, with a heart rate decrease below 6 bpm from peak effort for the 1^(st)^ minute of recovery, had a mortality rate > 20% at 1 year.

In line with these articles, Wagner et al.,^10^ reviewed the recommendations in the light of current evidence and classified pVO_2_, its percentage in relation to the maximum predicted pVO_2_ and the VE/CO_2_ slope as class I (LE A) recommendations, and the presence of OB as IIa (LE B) and OUES and P_ET_CO_2_ as IIb recommendations (LE B).

## Cardiac transplant indication: based on CPET and risk scores

The final decision to place a patient without contraindications on the HT waiting list is based on a risk-benefit analysis of the different therapeutic options, based on a clinical, psychological and social assessment, and of parameters provided by the complementary tests.

The CPET parameters can be considered separately or incorporated to scores such as HFSS and MECKI. The HFSS has seven variables, including pVO_2_. The MECKI, in turn, gives a higher weight to the CPET data when incorporated to the VE/VCO_2_ slope and the percentage of the maximum expected VO_2_ among its 5 variables.

Freitas et al.^11^ recently published an article comparing the value of 4 scores - HFSS, MECKI and two scores that integrate clinical parameters data: SHFM (10 variables) and MAGGIC (13 variables) - and MECKI was the most discriminative for CD or HT in the first year, with an area under the curve of 0.87.

## Conclusion

The CPET is indicated for risk stratification in HFREF, particularly in the assessment of candidates for HT and ventricular assistance, aiming to objectively quantify functional limitation and provide relevant clinical information on the etiology of functional limitations that may have a cardiac, pulmonary or mixed cause.^9^

It is not possible to perform CPET in patients in INTERMACS classes 1 to 3 (cardiogenic shock, receiving inotropic drugs or under circulatory assistance), in the presence of uncontrolled supraventricular or ventricular arrhythmias and in patients unable to exercise due to orthopedic pathology or extreme frailty.

However, in most patients in INTERMACS classes 4 to 7, provided that an exercise protocol adapted to the patient's functional capacity or an ergometer that allows minimizing their orthopedic limitations is selected, it is possible to perform a maximum CPET and obtain parameters with high prognostic value in most patients with HFREF.

Currently, pVO_2_, maximum predicted pVO_2_/VO_2_, VE/VCO_2_ slope and OB are considered as the parameters provided by the CPET with the highest prognostic value in HFREF.^9^

The CPET is still little used in Cardiology in the context of HF because its performance and interpretation involve some complexity, and because it has a higher cost than the conventional exercise test. However, it is of great interest as it allows an integrated assessment of the pathophysiology of the circulatory, respiratory and locomotor systems, making it possible to objectively identify the patients’ limitations, their cause, and stratify them in terms of prognosis.
